# Using a chatbot to reduce emergency department visits and unscheduled hospitalizations among patients with gynecologic malignancies during chemotherapy: A retrospective cohort study

**DOI:** 10.1016/j.heliyon.2023.e15798

**Published:** 2023-05-01

**Authors:** Ming-Yuan Huang, Chia-Sui Weng, Hsiao-Li Kuo, Yung-Cheng Su

**Affiliations:** a Department of Emergency, Mackay Memorial Hospital, Taipei, Taiwan; b Department of Medicine, MacKay Medical College, New Taipei City, Taiwan; c Department of Obstetrics and Gynecology, MacKay Memorial Hospital, Taipei, Taiwan; d School of Nursing, Chang Gung University, New Taipei City, Taiwan; e Department of Emergency Medicine, Ditmanson Medical Foundation Chia-Yi Christian Hospital, Chiayi City, Taiwan

**Keywords:** Chatbot, Patient-reported symptoms, Gynecologic malignancies

## Abstract

**Background:**

A chatbot is an automatic text-messaging tool that creates a dynamic interaction and simulates a human conversation through text or voice via smartphones or computers. A chatbot could be an effective solution for cancer patients' follow-up during treatment, and could save time for healthcare providers.

**Objective:**

We conducted a retrospective cohort study to evaluate whether a chatbot-based collection of patient-reported symptoms during chemotherapy, with automated alerts to clinicians, could decrease emergency department (ED) visits and hospitalizations. A control group received usual care.

**Methods:**

Self-reporting symptoms were communicated via the chatbot, a Facebook Messenger-based interface for patients with gynecologic malignancies. The chatbot included questions about common symptoms experienced during chemotherapy. Patients could also use the text-messaging feature to speak directly to the chatbot, and all reported outcomes were monitored by a cancer manager. The primary and secondary outcomes of the study were emergency department visits and unscheduled hospitalizations after initiation of chemotherapy after diagnosis of gynecologic malignancies. Multivariate Poisson regression models were applied to assess the adjusted incidence rate ratios (aIRRs) for chatbot use for ED visits and unscheduled hospitalizations after controlling for age, cancer stage, type of malignancy, diabetes, hypertension, chronic renal insufficiency, and coronary heart disease.

**Result:**

Twenty patients were included in the chatbot group, and 43 in the usual-care group. Significantly lower aIRRs for chatbot use for ED visits (0.27; 95% CI 0.11–0.65; p = 0.003) and unscheduled hospitalizations (0.31; 95% CI 0.11–0.88; p = 0.028) were noted. Patients using the chatbot approach had lower aIRRs of ED visits and unscheduled hospitalizations compared to usual-care patients.

**Conclusions:**

The chatbot was helpful for reducing ED visits and unscheduled hospitalizations in patients with gynecologic malignancies who were receiving chemotherapy. These findings are valuable for inspiring the future design of digital health interventions for cancer patients.

## Summary Points

• Patients using the chatbot had lower aIRRs of ED visits and unscheduled hospitalizations compared to those treated with usual care.
• Using chatbots as virtual consultants to deliver symptom interventions was effective in our study. This study implies the possible effectiveness of using a chatbot to gather patient-reported symptoms in cancer patients.

## 1 Introduction

Adverse side effects are common among patients receiving chemotherapy for gynecologic malignancies [1,2]. Usually, patients must manage their symptoms by themselves at home, and the lack of treatment adherence due to these side effects has been found to contribute to decreased survival rates [3,4]. Recently, patient-reported outcomes have been suggested as an approach to improve symptom control [5,6]. Several web-based systems have prompted clinicians to improve symptom management, communication, patient satisfaction, and better outcomes [2,7].

Information technology is on the rise, and is changing patient-physicians interactions. Technology-based self-service channels and digital health interventions have the potential to support patients with minimal time or with geographic restrictions, and to connect them to medical staff if necessary. The COVID-19 pandemic has further stimulated development of telemedicine and the use of digital platforms [8]. Among these platforms, a chatbot is an automatic text-messaging tool that communicates information to patients based on questions they submit [9]. These tools create a dynamic interaction, are easy to use, and simulate human conversation through text or voice via smartphones or computers. A chatbot could be an effective solution for cancer patients' follow-up during treatment by improving their quality of life. Use of a chatbot could also save time for healthcare providers. Using the chatbot, patients can interact to describe their symptoms; in return, chatbots provide professional problem-solving advice and information to patients [9,10].

While it seems likely that patients will find the chatbot useful to address symptoms and to seek consultations, it is not known if unnecessary emergency department (ED) visits and unscheduled hospitalizations are potentially avoidable through improved prospective monitoring and symptoms management. To address these questions, we conducted a retrospective cohort study to evaluate whether a chatbot-based collection of patient-reported symptoms during chemotherapy treatment, with automated alerts to clinicians for severe or worsening symptoms, decreases ED use and reduces unscheduled hospitalizations.

## 2 Methods

### 2.1 Chatbot design and function

To develop the chatbot, we reviewed online resources of questions/answers and assessed health instruction materials for patients receiving chemotherapy from our hospital as well as other health institutions. We also interviewed cancer managers, nurses, and physicians, to verify the appropriateness and accuracy of using these educational materials in a chatbot design. A multidisciplinary design team constructed the patient interface and user experience for the chatbot. To use the chatbot, patients were instructed to scan a QR-code-embedded link for the chatbot. They were immediately redirected to a Facebook Messenger, where the chatbot was built in. During the course of chemotherapy, the chatbot interacts with patients every day to ask their current condition and to instantly provide relevant instructions. The chatbot software also uses intelligence and empathy to engage with users. To facilitate the conversation, the chatbot agent begins with a greeting combined with an emotion assessment and a quick follow-up response for mental health support. The chatbot agent soon moves on to following main phases, which includes (1) acquisition of a follow-up on patients' daily conditions, (2) sequential question-and-answering for symptoms assessment, (3) information extraction by medical entity recognition, and (4) natural language generation to user responses based on pre-defined templates of health instructions.

### 2.2 Study design and participants

Patients starting chemotherapy for gynecologic cancer at MacKay Memorial Hospital (MMH), Taipei, Taiwan, during January 1, 2020 to January 31, 2021, were introduced to the digital health innovative project. The case managers invited the patients to enroll in the chatbot program after their first chemotherapy session. Joining such a service is not mandatory, but usually if patients were confident about their online digital capabilities, had internet support, and were accustomed to using social software, they could use the chatbot.

We identified a convenience sample of 20 adult patients for chatbot self-reporting of symptoms, and compared this with a group of 43 adult patients receiving usual care. This retrospective cohort study was approved by the Institutional Review Board of MMH.

### 2.3 Intervention

Self-reporting symptoms were conducted via the chatbot, a Facebook Messenger-based interface established as easy to use for patients with cancer and symptom burdens. The chatbot includes questions about common symptoms experienced during chemotherapy [1,2], such as vomiting, nausea, loss of appetite, oral mucositis, diarrhea, peripheral neuropathy, palmar plantar erythrodysesthesia, fatigue, maculopapular rash, constipation, and insomnia. These symptoms are graded based on the National Cancer Institute’s Common Terminology Criteria for Adverse Events (CTCAE) [11]. To make it easier for patients to respond, we modified the CTCAE criteria into a 4-point scale: 0 (not present), 1 (grade 1), 2 (grade 2), and 3 (grades 3, 4, and 5 (Video 1). If patients experienced symptoms other than these, they could also directly use the text message function to talk to the chatbot (Video 2). All patients' report outcomes were monitored by a cancer manager.

Supplementary data related to this article can be found at https://doi.org/10.1016/j.heliyon.2023.e15798.

The following are the supplementary data related to this article:

Multimedia component 1 The interface of patient-reported symptoms Multimedia component 1

Multimedia component 2 Communication with the chatbot via text message Multimedia component 2

At enrollment, the chatbot sends notifications to patients regarding the patients’conditions. The patients then respond to the chatbot based on the symptoms they have, and the chatbot will send prearranged suggestions about how to relieve these symptoms directly to the patients. At the end of the conversations, patients were also asked to evaluate and note if the conversations were helpful.

If patients presented with disabling conditions (more than CTCAE grade 3) or if the chatbot software was unable to respond to a patient’s questions, the cancer manager contacted the patient to initiate a phone or online discussion, and to provide any direct help if needed.

### 2.4 Usual care

Usual care consisted of the standard care procedures at MMH for oncology patients during their chemotherapy sessions. With standard care, symptoms are discussed and documented in the medical record during clinical encounters between patients and their oncologists. Patients with direct concerns about symptoms were also encouraged to initiate telephone contact with cancer managers between visits.

### 2.5 Statistical analyses

Age and follow-up year were classified as continuous variables, and all other covariates were classified as categorical variables. To evaluate baseline heterogeneity between the two study groups, categorical and continuous variables were compared using Fisher’s exact test and a *t*-test, respectively. The contents of the chatbot usage were also described.

The primary outcome of the study was the number of ED visits after initiation of chemotherapy after diagnosis of gynecologic malignancies. We first evaluated the rate of ED visits per patient-years overall between the two groups. Follow-up time was determined based on the date of diagnosis to the end of the study period (January 31, 2021) or death. Since the outcomes are the count data observed over a period of follow-up, we applied the multivariate Poisson model to evaluate adjusted incidence rate ratios (aIRRs) of ED visits, using follow-up time as an offset after recording age, cancer staging, type of malignancy, and presence of diabetes, hypertension, chronic renal insufficiency, and coronary heart disease. Age and cancer staging were recorded as continuous variables in this model.

The secondary outcomes were the unscheduled hospitalizations after initiation of chemotherapy after diagnosis. We defined unscheduled hospitalizations as hospitalizations during the follow-up period, excluding in-hospital chemotherapy. We applied the multivariate Poisson model to evaluate the aIRRs of numbers of admissions after adjusting for relevant factors mentioned above. All p-values were two-tailed, and p < 0.05 was considered statistically significant. All analyses were performed using SAS™ Statistical Analysis Software for Windows, V.9.4 (SAS Institute, Cary, NC, USA).

## 3 Results

Table 1 summarizes the demographic characteristics and comorbidities of the chatbot group (n = 20) and the usual-care group (n = 43). One patient in the usual-care group died during the study period. Patients in the chatbot group were younger than the usual-care group, (47.75 years of age vs. 63.67 years of age, respectively), while the comorbidities and cancer staging were similar between the two groups. The total follow-up times were 11.72 (chatbot group) and 23.34 (usual-care group) person-years, respectively. At the end of the follow-up period, there were 52 total episodes of ED visits (8 in the chatbot group and 44 in the usual care group), making the incidence rates 0.68 and 1.89 per person-years, respectively.

**Table 1:** Baseline characteristics of the chatbot group and the usual care group.

Variables	Chatbot group (n = 20)		Usual care group (n = 43)		p-value
	Number	% or SD	Number	% or SD	
Mean follow-up year (SD)	0.59	0.25	0.54	0.23	0.495
Mean ED visits (SD)	0.4	0.5	1.02	1.26	0.007
Mean age (SD)	47.75	8.91	63.67	11.06	<0.001
Cancer staging					
I	8	40	17	39.53	0.536
II	1	5	0	0	
III	6	30	16	37.21	
IV	5	25	10	23.26	
Cancer type					
Endometrial cancer	6	30	14	32.56	1
Ovarian cancer	14	70	29	67.44	
Diabetes	0	0	6	13.95	0.09
Hypertension	3	15	11	25.58	0.518
Chronic renal insufficiency	0	0	4	9.3	0.298
Mortality	0	0	1	2.33	1

**Fig. 1:**
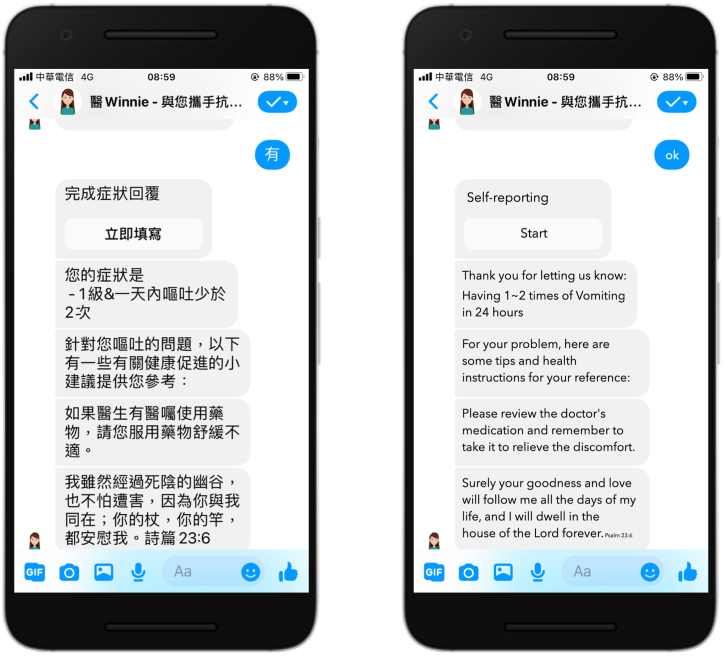
A screenshot of the chatbot interface.

A total of 897 consultations were recorded during the study period. Fig. 1 shows a screenshot of chatbot use. Most patients reported symptoms of 0–1 on the CTCAE criteria scale. Based on the data, up to 1.56% of consultations required further in-person communication. Most patients (97.24%) were satisfied with the automated responses and suggestions from the chatbot. Information about patient-reported symptoms via chatbot conversation contents is summarized in Table 2.

**Table 2:** Information about patient-reported symptoms via chatbot.

Symptoms	Scales	Definitions	N	%
Vomiting	0	No vomiting	882	98.33
	1	1-2 episodes of vomiting within 24 h; no intervention needed	14	1.56
	2	3-5 episodes of vomiting within 24 h; needs medical attention	1	0.11
	3	≥6 episodes of vomiting within 24 h; needs hospitalization	0	0
Nausea	0	No nausea	788	87.85
	1	Loss of appetite without alteration in eating habits	101	11.26
	2	Oral intake decreased without significant weight loss, dehydration, or malnutrition	2	0.22
	3	Inadequate oral caloric or fluid intake; tube feeding, TPN, or hospitalization indicated	6	0.67
Appetite loss	0	No anorexia	633	73.91
	1	Loss of appetite without change in eating habits	199	22.19
	2	Oral intake altered without significant weight loss or malnutrition; oral nutritional supplements indicated	27	3.01
	3	Associated with significant weight loss or malnutrition (e.g., inadequate oral caloric and/or fluid intake); tube feeding or TPN indicated	8	0.89
Mucositis	0	No mucositis	832	92.75
	1	Mild symptoms; intervention not indicated	44	4.91
	2	Moderate pain or ulcer that does not interfere with oral intake; modified diet indicated	7	0.78
	3	Bleeding or severe pain of oral ulcer; interferes with oral intake	14	1.56
Diarrhea	0	No diarrhea	819	91.3
	1	Increase of <4 stools per day over baseline; mild increase in ostomy output compared to baseline	61	6.8
	2	Increase of 4–6 stools per day over baseline; moderate increase in ostomy output compared to baseline; limiting instrumental ADL	11	1.23
	3	Increase of ≥ 7 stools per day over baseline; hospitalization indicated; severe increase in ostomy output compared to baseline; limiting self-care ADL	6	0.67
Peripheral motor neuropathy	0	No peripheral motor neuropathy	640	71.35
	1	Mild symptoms; no limiting ADL	256	28.54
	2	Moderate symptoms; limiting instrumental ADL	1	0.11
	3	Severe symptoms; limiting self-care ADL		
Palmar-plantar erythrodysesthesia syndrome (PPE)	0	No PPE	870	96.99
	1	Minimal skin changes or dermatitis (e.g., erythema, edema, or hyperkeratosis) without pain	20	2.23
	2	Skin changes (e.g., peeling, blisters, bleeding, fissures, edema, or hyperkeratosis) with pain, limiting instrumental ADL	5	0.56
	3	Severe skin changes (e.g., peeling, blisters, bleeding, fissures, edema, or hyperkeratosis) with pain, limiting self-care ADL	2	0.22
Fatigue	0	No fatigue	480	53.51
	1	Fatigue relieved by rest	354	39.46
	2	Fatigue not relieved by rest, limiting instrumental ADL	61	6.8
	3	Fatigue not relieved by rest, limiting self-care ADL	2	0.22
Erythema multiforme	0	No erythema multiforme	818	91.19
	1	Target lesions covering <10% BSA and not associated with skin tenderness	75	8.36
	2	Target lesions covering 10%–30% BSA and associated with skin tenderness, limiting instrumental ADL	4	0.45
	3	Target lesions covering >30% BSA and associated with oral or genital erosions, limiting self-care ADL		
Constipation	0	No constipation	685	76.37
	1	Occasional or intermittent symptoms; occasional use of stool softeners, laxatives, dietary modification, or enemas	154	17.17
	2	Persistent symptoms with regular use of laxatives or enemas; limiting instrumental ADL	58	6.47
	3	Obstipation with manual evacuation indicated; limiting self-care ADL	0	0
Insomnia	0	No insomnia	667	74.36
	1	Mild difficulty falling asleep, staying asleep, or waking up early	180	20.07
	2	Moderate difficulty falling asleep, staying asleep, or waking up early	47	5.24
	3	Severe difficulty in falling asleep, staying asleep or waking up early	3	0.33

Key: ADL = activities of daily living; BSA = body surface area; TPN = total parenteral nutrition.

We then applied a multivariate Poisson regression model to assess the aIRRs for chatbot use for ED visits, after controlling for the above-mentioned covariates (Table 3). The results show a significantly lower aIRR for chatbot use for ED visits (0.27; 95% CI 0.11–0.65; p = 0.003). The other factors independently associated with increased ER visits were younger age, more advanced cancer stage, and diabetes.

**Table 3:** Adjusted incidence rate ratios (aIRRs) of ED visits.

Variables	aIRR	95% Confidence interval		p-value
Chatbot group	0.27	0.11	0.65	0.003*
Age (every one-year increase)	0.97	0.95	1	0.042*
Cancer stage (every one-stage increase)	1.44	1.1	1.88	0.008*
Endometrial cancer vs. cervical cancer	0.78	0.42	1.47	0.451
Diabetes	2.14	1.03	4.44	0.043*
Hypertension	0.79	0.32	1.92	0.595
Chronic renal insufficiency	0.75	0.2	2.83	0.672

∗ Statistically significant

As for unscheduled hospitalizations, there were 6 episodes in the chatbot group and 30 in the usual-care group, respectively. We further analyzed the aIRRs of unscheduled hospitalizations between the two groups after applying the multivariate Poisson regression mentioned before. The results reveal a significantly lower aIRR for chatbot use for unscheduled hospitalizations (0.31; 95% CI 0.11–0.88; p = 0.028). The results are summarized in Table 4.

**Table 4:** Adjusted incidence rate ratios (aIRRs) of unscheduled hospitalizations.

Variables	aIRR	95% confidence interval		p-value
Chatbot group	0.31	0.11	0.88	0.028*
Age (every one-year increase)	0.98	0.95	1.01	0.277
Cancer stage (every one-stage increase)	1.35	0.99	1.86	0.062
Endometrial cancer vs. cervical cancer	1.15	0.57	2.33	0.691
Diabetes	1.28	0.51	3.22	0.607
Hypertension	0.89	0.30	2.64	0.828
Chronic renal insufficiency	1.25	0.30	5.24	0.758

∗ Statistically significant

## 4 Discussion

Patients using the chatbot approach had lower aIRRs of ED visits and unscheduled hospitalizations compared to patients treated with usual care. The mortality rates were similar between the two groups, indicating that fewer ED visits and admissions did not result in poorer prognoses during the study period. The use of chatbot-assisted cancer care may decrease the medical interventions and expenses. The feedback from the chatbot users was positive, further revealing a promising future of this integrated care for patients.

Patient-reported symptoms have the potential to narrow the gap in clinical manifestations observed between healthcare providers and patients [12,13]. The clinical benefits may come from increased rates of symptom discussions between patients and healthcare providers, intensified symptom management based on patient reports, and improved symptom control when patient reports are shared with clinicians [2,14,15]. In our study, use of the chatbot led to a significant reduction of ED visits and unscheduled hospitalizations. These findings are in line with recent studies targeting cancer populations [2,[16], [17], [18]]. Using chatbots as virtual consultants to deliver symptom interventions was effective in our study [19,20]. However, the data regarding the use of chatbots in oncology are still limited, unlike their potential benefits for other patients and the healthcare system [10]. To our knowledge, this study is also one of the first to utilize the chatbot as a bridge between non-English-speaking cancer patients and healthcare providers.

Increased popularity of digital technology and availability of mobile phones have offered the possibility of delivering digital health interventions in a more convenient way [21,22]. These solutions seem to be particularly appropriate to deploy in the time of the current COVID-19 pandemic, when reducing unnecessary ED visits and hospitalizations can decrease the possibility of iatrogenic infection among these relatively immunocompromised patients [10]. Chatbots may also save patients with minor symptoms from an unneeded hospital visit, which could allow healthcare providers to spend more time treating patients who need treatment the most, further saving money and resources.

## 5 Limitations

Our study had several limitations. First, it was conducted at a single urban medical center, and thus the generalizability may be limited. Second, because it was a pilot digital health program, we did not arrange the randomization in the two groups. The patients in the intervention group were younger and thus might be more open-minded toward and familiar with, digital tools, which may have led to sampling bias. Differences in age and diabetes between the two groups may raise the possibility of confounding even after statistical adjustments. A randomized controlled study should be conducted to evaluate the efficacy of the chatbot. Third, in our study, the chatbot was designed to respond with suggestions only when the symptoms reported by the patients were mild. We did not design the chatbot to replace healthcare providers; instead, we consider it an enhancement to usual cancer care. By doing so, the ED visits and unscheduled hospitalizations were significantly reduced, and the overall satisfaction from patients was high. Finally, the number of patients enrolled in our project was relatively small, and limited statistical power may be an issue. If we performed the matched analysis to reduce the heterogeneity then the numbers in the two subgroups will be too small, which may further introduce type 2 error in our study. We acknowledge we could enroll more patients in the unexposed group for statistical flexibility by extending the study period. However, the year 2020 is the COVID-19 pandemic, and the ED visits are decreased during this year in Taiwan. If we enrolled patients as unexposed group from other period, we may not be able to compare the two groups under the same baseline condition. We are planning a larger-scale study involving a variety of cancer patients, to further evaluate the benefits of chatbot use in cancer patients other than those with gynecologic malignancies.

## 6 Conclusions

This study further extends previous research on patient-reported symptoms in cancer patients, implying the possible effectiveness of the chatbot in reducing ED visits and unscheduled hospitalizations in patients with gynecologic malignancies who are receiving chemotherapy. These findings may be valuable for inspiring the future design of digital health interventions for cancer patients.

## Declarations

### Ethics approval and consent to participate

This study was initiated after approval from the Institutional Review Board of Taipei Mackay Memorial Hospital, Taiwan.

### Consent for publication

Not applicable.

### Data sharing statement

The datasets used and analyzed for the current study are available from the corresponding author on reasonable request.

## Declaration of competing interest

The authors declare that they have no known competing financial interests or personal relationships that could have appeared to influence the work reported in this paper
